# Efficacy of liraglutide 3.0 mg treatment on weight loss in patients with weight regain after bariatric surgery

**DOI:** 10.1007/s40519-022-01403-9

**Published:** 2022-06-28

**Authors:** Fabrizio Muratori, F. Vignati, G. Di Sacco, L. Gavazzi, D. Pellegrino, M. Del Prete

**Affiliations:** 1 Division of Endocrinology and Diabetology, Sant’Anna Hospital–ASST Lariana, Como, Italy; 2 Division of Geriatrics, Sant’Anna Hospital–ASST Lariana, Como, Italy

**Keywords:** Liraglutide, Obesity, Weight regain, Bariatric surgery

## Abstract

**Purpose:**

Bariatric surgery, as Roux-en-Y gastric bypass (RYGB), laparoscopic gastric banding (LGB), and laparoscopic sleeve gastrectomy (LSG), is considered the gold standard treatment to achieve long-term weight loss in severe obesity. In patients who fail to maintain the achieved weight, pharmacological treatment may be required. Here, we reported our real-life experience on the efficacy of liraglutide therapy in 62 patients who regained weight after bariatric surgery.

**Methods:**

We retrospectively evaluated 62 (60 F-2 M; mean age: 43.6 ± 9.9 years) patients received liraglutide for weight loss after bariatric surgery (17 RYGB, 22 LGB, and 23 LSG). Body mass index (BMI) before and after surgery was, respectively, of 45.4 ± 5.5 kg/m^2^ and 29.5 ± 4.9 kg/m^2^. Patients were followed up from 2016 until 2021. Liraglutide was administered after weight regain once-daily subcutaneously at starting dose of 0.6 mg and with weekly increases up to 3.0 mg. Treatments were administered when a weight regain of 10–15% occurred after reaching a minimum weight loss from bariatric surgery or if weight loss after bariatric surgery was unsatisfactory.

**Results:**

After a mean of 70.7 ± 43.7 months from any bariatric surgery, all patients started liraglutide therapy. At this time, mean BMI was 34.2 ± 4.8 kg/m^2^ (mean increased BMI: 4.7 ± 2.8 kg/m^2^). After a mean of 10.5 ± 4.4 months from the beginning of liraglutide, 9 patients achieved normal weight (BMI 24.1 ± 0.9 kg/m^2^), and 28 were overweight (BMI 26.9 ± 1.6 kg/m^2^). Twenty patients achieved grade I (BMI 32.1 ± 1.5 kg/m^2^), 5 grade II (BMI 37.3 ± 2.0 kg/m^2^) obesity, and none had grade III obesity (mean BMI change: − 5.1 ± 2.5 kg/m^2^). The treatment was well tolerated, and no serious adverse events were recorded.

**Conclusion:**

These data confirm the efficacy and safety of liraglutide in patients who experienced weight regain after bariatric surgery. Considering the long-term follow-up, patients should be followed up regularly and the pharmacological treatment should be adapted to the weight fluctuations observed during the clinical history.

**Level of evidence:**

V. Opinions of authorities, based on descriptive studies, narrative reviews, clinical experience, or reports of expert committees.

## Introduction

Bariatric surgery is considered as the gold standard treatment to achieve significant and durable weight loss in severe obesity, especially in patients with severe complications as diabetes mellitus, sleep apnea syndrome, or patients with long experience of weight cycling [1, 2].

The Swedish Obese Subjects (SOS) study first showed a permanent weight loss after 15-year follow-up and a reduction in mortality as well as the incidence of type 2 diabetic disease and some kind of cancer in patients undergoing bariatric surgery [3, 4]. These results were confirmed by subsequent studies [5, 6].

Although the weight loss observed after bariatric surgery is 30–45%, about 10–20% of patients undergoing these procedures experienced weight regain or insufficient weight loss [1, 2]. However, in the literature, there is no standard definition referring to the percentage of weight regain after bariatric surgery. Studies usually report the pre-bariatric weight, the maximum weight loss after surgery, and the weight regained years later [7].

It has been reported that 5 years after Roux-en-Y gastric bypass (RYGB) surgery, about 1/4 of patients have regained 15% of their weight from the nadir after surgery and 2/3 have regained about 20% [7]. Some studies have tried to identify the reasons for weight regain after bariatric surgery. The reasons for weight regain are many such as the poor adherence to nutritional advice during the follow-up after bariatric surgery [8–10], an increase in ghrelin in about 20% of surgically treated patients [11–13], inadequate post-surgery physical activity [10], and not well controlled psychiatric disorders [14]. Furthermore, some authors have observed how the dilation of the gastric stoma post-gastric bypass can have an influence on weight regain [15]. Recently, liraglutide 3.0 mg has been approved for the treatment of obesity. In phase III studies, liraglutide has been shown to be effective not only in increasing weight loss in a program including a calorie-restricted nutritional plan, but also in maintaining weight after weight loss [16–18].

Given the increasing number of bariatric surgeries, weight gain can adversely affect metabolic complications. Liraglutide can therefore be used in those patients undergoing bariatric surgery who have not lost enough weight or who have partially regained the weight lost [6, 19, 20].

In this retrospective study, we report the efficacy and safety of liraglutide in 67 patients who had previously undergone bariatric surgery followed for a period of 6–48 months. It is, therefore, necessary to identify a therapeutic strategy to treat weight gain after bariatric surgery.

## Materials and methods

From 2016 to 2020, 62 non-diabetic obese patients were retrospectively evaluated for weight loss with liraglutide after any bariatric surgery at the Sant’Anna Hospital of Como to assess the efficacy and safety of liraglutide treatment in this setting of patients. Patient characteristics are presented in Tables 1 and 2. Of the 62 enrolled patients, 50 spontaneously presented in our outpatient clinic, coming from other centers where they had been operated on, while 12 patients were referred to the surgeon by our team and subsequently followed up in a regular post-surgery follow-up.

**Table 1:** Patient characteristics

	Patients (*n* = 62)
Age (years; mean ± SD)	43.6 ± 9.9
Sex (*n*)	
F	60
M	2
Previous surgery (*n*)	
RYGB	17
LGB	22
LSG	23
Baseline BMI (kg/m^2^; mean ± SD)	
Before surgery	45.4 ± 5.5
After surgery	29.5 ± 4.9
Before liraglutide	34.2 ± 4.8
Time between surgery and liraglutide therapy (months; mean ± SD)	70.7 ± 43.7

*RYGB* Roux-en-Y gastric bypass, *LGB* laparoscopic gastric banding, *LSG* laparoscopic sleeve gastrectomy, *BMI* body mass index, *SD* standard deviation

**Table 2:** Detailed patient characteristics

Patients	Sex	Age at first visit	Surgery	BMI				Time between surgery and liraglutide onset (months)	Duration of liraglutide therapy (months)
				Before surgery	After surgery	Before liraglutide	After liraglutide		
1	F	47	LGB	47.0	30.4	32.5	25.8	48	6
2	F	44	LGB	42.5	40.8	42.5	25.5	24	8
3	F	47	LSG	48.8	27.3	39.1	26.6	72	6
4	F	22	LGB	41.8	34.5	35.7	34.0	24	12
5	F	49	LGB	45.0	31.3	38.2	24.5	48	8
6	F	53	LGB	43.4	40.6	42.6	28.8	12	6
7	F	31	LGB	41.5	30.7	33.2	25.8	36	12
8	F	35	LGB	47.1	34.9	40.4	28.1	48	10
9	F	46	LGB	44.6	31.6	35.1	30.1	24	6
10	F	45	LGB	41.8	31.3	34.0	22.1	24	7
11	F	32	LGB	40.5	37.4	38.8	24.3	48	14
12	F	57	LSG	45.5	28.0	32.1	31.0	48	12
13	F	24	LSG	40.1	23.6	30.4	24.9	132	6
14	F	31	LSG	53.3	32.9	40.0	24.2	60	18
15	F	48	LSG	36.2	20.2	27.7	34.4	48	6
16	F	55	RYGB	45.4	26.6	34.0	26.8	84	18
17	F	38	LGB	46.3	38.2	43.0	33.6	60	17
18	F	38	LGB	50.9	32.7	41.8	26.9	132	16
19	F	28	LSG	41.6	26.3	30.5	27.2	48	12
20	F	55	RYGB	53.9	31.3	38.2	34.2	60	18
21	F	55	LGB	42.3	19.1	26.5	31.1	120	5
22	F	44	LSG	46.6	31.2	32.5	25.8	60	18
23	F	47	LGB	47.4	31.2	34.1	25.5	96	18
24	F	63	LGB	38.8	23.3	32.2	26.6	144	4
25	F	47	LGB	42.6	37.5	42.5	34.0	48	8
26	F	54	LSG	35.9	23.8	29.1	24.5	84	8
27	F	58	LSG	50.8	30.9	36.5	28.8	72	12
28	F	28	LSG	57.6	39.3	40.5	25.8	30	8
29	F	61	LGB	41.5	31.1	36.7	28.1	120	18
30	F	39	LSG	37.5	28.4	30.1	30.1	36	8
31	F	49	LSG	41.0	25.7	34.9	22.1	48	13
32	F	49	LSG	41.9	27.2	39.3	24.3	72	8
33	F	44	LGB	52.8	31.9	38.3	31.0	108	14
34	F	54	LGB	44.1	25.7	29.9	24.9	108	10
35	F	40	LSG	41.5	31.7	34.7	24.2	36	6
36	F	31	LGB	42.7	28.5	31.3	34.4	60	12
37	F	39	LSG	51.5	29.3	33.2	26.8	36	7
38	F	53	LGB	42.8	27.4	33.6	33.6	144	10
39	F	23	LGB	40.2	22.2	29.1	26.9	216	10
40	F	44	LSG	41.5	24.2	35.1	27.2	48	12
41	F	52	LSG	41.3	24.6	27.1	34.2	24	4
42	F	48	LSG	42.7	24.4	27.6	31.1	144	3
43	F	44	LSG	40.3	25.5	28.4	25.8	72	6
44	F	48	LSG	43.4	26.6	29.3	25.5	24	6
45	F	41	LSG	55.8	32.8	38.5	26.6	36	6
46	M	41	LSG	35.1	25.5	28.4	34.0	24	8
47	F	26	LSG	49.2	29.6	34.0	24.5	37	12
48	F	26	RYGB	44.9	25.5	29.8	28.8	96	14
49	F	44	RYGB	46.9	27.3	31.6	25.8	108	12
50	F	50	RYGB	52.2	31.0	35.3	28.1	30	7
51	M	49	RYGB	40.3	25.5	26.4	30.1	60	12
52	F	45	RYGB	47.8	25.7	28.7	22.1	60	12
53	F	50	RYGB	50.7	32.5	38.0	24.3	36	16
54	F	45	RYGB	49.4	26.1	31.7	31.0	156	18
55	F	50	RYGB	43.1	24.2	25.8	24.9	30	3
56	F	32	RYGB	46.3	30.9	41.8	24.2	72	14
57	F	30	RYGB	49.7	29.7	29.7	34.4	30	7
58	F	51	RYGB	58.6	34.4	38.3	26.8	144	12
59	F	51	RYGB	47.0	27.6	32.1	33.6	132	16
60	F	44	RYGB	45.1	27.6	30.2	26.9	120	12
61	F	49	RYGB	59.6	39.3	41.5	27.2	120	18
62	F	41	RYGB	47.8	31.8	35.3	34.2	60	8

RYGB = Roux-en-Y gastric bypass; laparoscopic gastric banding (LGB); LSG = laparoscopic sleeve gastrectomy; BMI = body mass index

Patients were aged > 18 years and started liraglutide when there was a 10–15% weight regain over the nadir obtained after any bariatric surgery [19]. Some patients with weight regain of less than 10% after bariatric surgery spontaneously asked to be treated and were also included in this retrospective study. Bariatric surgeries performed were Roux-en-Y gastric bypass (RYGB), laparoscopic gastric banding (LGB), and laparoscopic sleeve gastrectomy (LSG), respectively, in 17, 22, and 23 patients.

Before starting liraglutide, patients were assessed to exclude any condition that contraindicate the therapy. Pregnancy and breastfeeding were also excluded.

Liraglutide was administered after weight regain once-daily subcutaneously at starting dose of 0.6 mg and with weekly increases up to 3.0 mg. Data on liraglutide efficacy and safety were recorded. The efficacy was assessed by calculating the percentage of weight loss after liraglutide treatment as mean BMI change in all patients and subgroups according to the degree of obesity. Liraglutide treatment was administered until patients achieved a minimum weight loss after bariatric surgery of 10–15% and was administered again when weight regain of 10–15% occurred to get the desired weight loss. Patient consents were obtained before starting the study.

Data are presented as the mean ± SD. Two-tailed paired *t* tests were used for comparing normally distributed data concerning BMI changes. A difference was considered statistically significant if the corresponding *p* value was < 0.05. Data were analyzed using GraphPad Prism software (version 9, GraphPad Software Inc., La Jolla, CA).

## Results

After a mean of 70.7 ± 43.7 months from any bariatric surgery, 62 patients started therapy with liraglutide (Tables 1, 2). At this time, mean BMI was 34.2 ± 4.8 kg/m^2^ with an overall mean increased BMI of 4.7 ± 2.8 kg/m^2^ (mean increased BMI of 4.5 ± 2.4 kg/m^2^ for LGB, 5.2 ± 3.3 kg/m^2^ for LSG, and 4.2 ± 2.6 kg/m^2^ for RYGB). Fifteen patients were overweight (BMI 28.0 ± 1.2 kg/m^2^), 22 had grade I (BMI 32.4 ± 1.6 kg/m^2^), 15 grade II (BMI 37.2 ± 1.6 kg/m^2^), and 10 grade III (BMI 41.7 ± 1.2 kg/m^2^) obesity.

At baseline in the LGB group, 2 patients were overweight (BMI 27.8 ± 1.8 kg/m^2^), 8 grade I (BMI 32.6 ± 1.4 kg/m^2^), 6 grade II (BMI 37.1 ± 1.5 kg/m^2^), and 6 grade III (BMI 42.1 ± 0.9 kg/m^2^) obesity. In the LSG group, 7 patients were overweight (BMI 28.2 ± 0.8 kg/m^2^), 9 grade I (BMI 32.5 ± 1.9 kg/m^2^), 5 grade II (BMI 37.7 ± 1.8 kg/m^2^), and 2 grade III (BMI 40.3 ± 0.4 kg/m^2^) obesity. In the RYGB, 5 patients were overweight (BMI 28.1 ± 1.9 kg/m^2^), 5 grade I (BMI 31.9 ± 1.4 kg/m^2^), 5 grade II (BMI 37.0 ± 1.6 kg/m^2^), and other 2 patients grade III (BMI 41.6 ± 0.2 kg/m^2^) obesity.

**Fig. 1:**
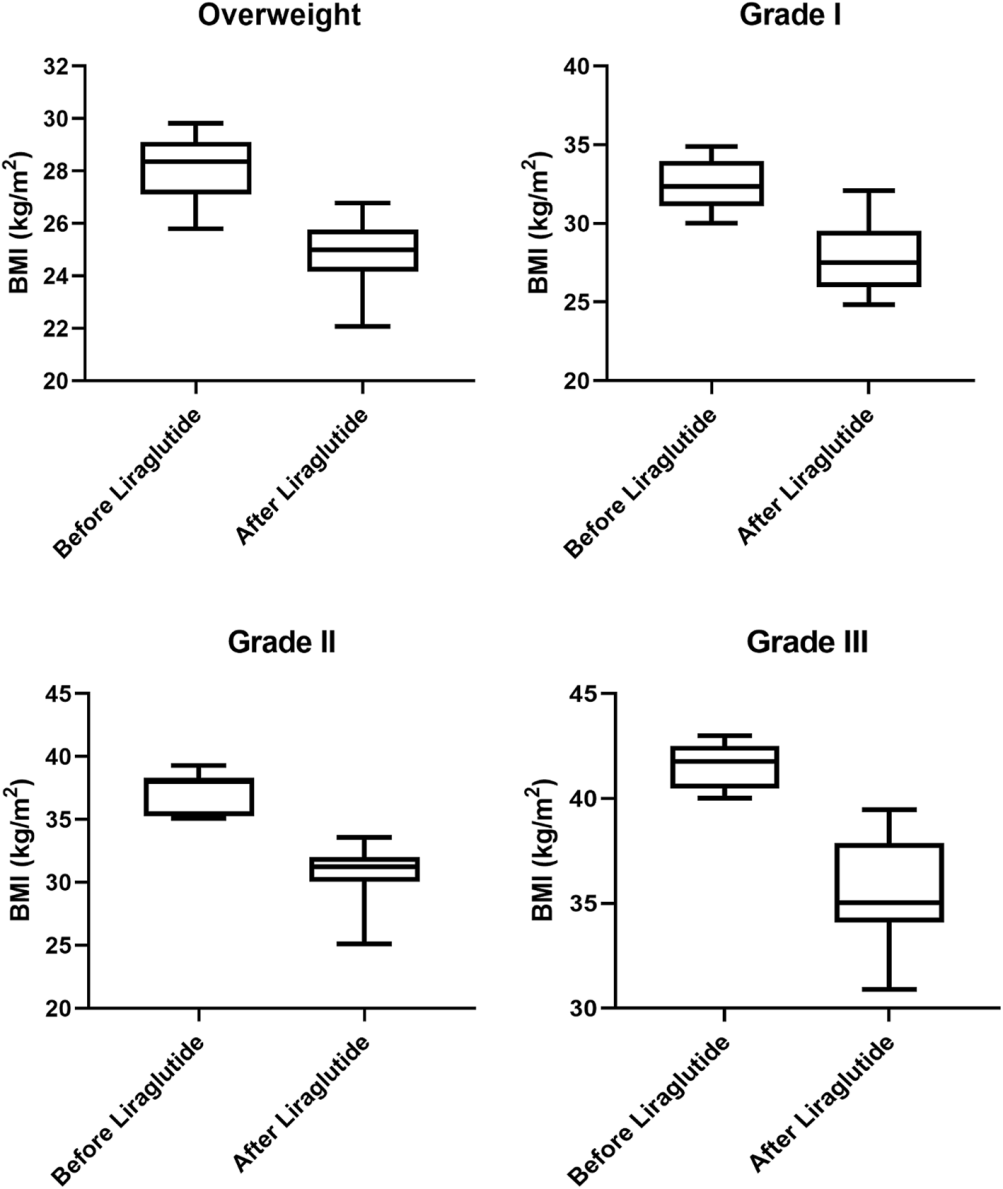
Body mass index (BMI) changes in patients treated with liraglutide according to the obesity grade. Boxes represent the interquartile range, and whiskers represent the interdecile range

After a mean of 10.5 ± 4.4 months from the beginning of liraglutide therapy, ranging from 1 to 3 cycles, mean BMI was significantly reduced in all patients (*p* < 0.0001). Nine patients achieved normal weight (BMI 24.1 ± 0.9 kg/m^2^) and 27 overweight (BMI 26.9 ± 1.6 kg/m^2^). Twenty-one patients achieved grade I (BMI 32.1 ± 1.5 kg/m^2^), 5 grade II (BMI 37.3 ± 2.0 kg/m^2^) obesity, and none had grade III obesity (mean BMI change: − 5.1 ± 2.5 kg/m^2^) (Fig. 1).

Considering each type of surgery, after liraglutide treatment, in the LGB group, 2 patients achieved normal weight (BMI 23.7 ± 0.7 kg/m^2^), 8 overweight (BMI 27.8 ± 1.8 kg/m^2^), 7 grade I (BMI 31.4 ± 0.9 kg/m^2^), and 5 grade II (BMI 37.3 ± 2.0 kg/m^2^) obesity. In the LSG group, 2 patients had normal weight (BMI 24.5 ± 0.0 kg/m^2^), 14 overweight (BMI 26.4 ± 1.4 kg/m^2^), and 7 grade I obesity (BMI 32.9 ± 1.3 kg/m^2^), while in the RYGB, 5 patients had normal weight (BMI 24.0 ± 1.1 kg/m^2^), 5 overweight (BMI 27.0 ± 0.9 kg/m^2^), and 7 grade I obesity (BMI 32.1 ± 1.9 kg/m^2^).

**Fig. 2:**
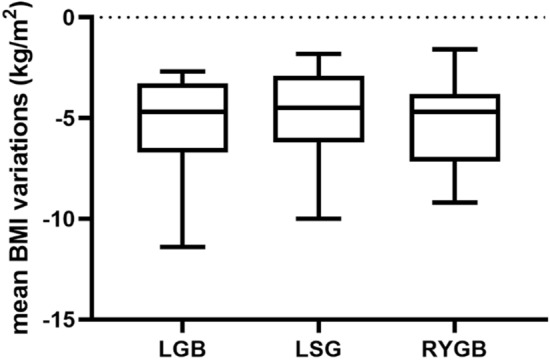
Comparison of mean body mass index (BMI) variations after liraglutide therapy in patients treated with liraglutide according to the type of bariatric surgery. Boxes represent the interquartile range, and whiskers represent the interdecile range

By comparing the mean BMI variations of each subgroup of patients according to the type of bariatric surgery, no significant differences were observed, indicating a significant weight loss regardless of the type of bariatric surgery performed (LGB vs LSG *p* = 0.4895; LGB vs RYGB *p* = 0.9618; LSG vs RYGB *p* = 0.5053) (Fig. 2).

The treatment was well tolerated, and no serious adverse events (AE) were recorded. Seven patients experienced mild AE as nausea and vomiting from the beginning of the treatment. All patients had resolution of these events with liraglutide dose adjustment, and no patients discontinued the treatment for safety reasons. No other AE event was reported.

## Discussion

Our results confirm, in line with the other studies, that the use of anti-obesity drugs in patients with weight regain or insufficient weight loss after bariatric surgery is effective and should be considered. In our study, liraglutide proved to be particularly effective in all three considered surgeries: laparoscopic gastric banding (LGB), laparoscopic sleeve gastrectomy (LSG), and Roux-en-Y gastric bypass (RYGB).

In the current study, of the 62 enrolled patients, 50 spontaneously presented in our outpatient clinic, coming from other centers where they had been operated on. Other 12 patients had been referred to the surgeon by our team and were subsequently followed up in a regular post-surgery follow-up. From the studies published up to now, there is no precise clinical reference parameter to decide when to start drug therapy in patients with weight regain after bariatric surgery.

In the 12 subjects we sent for surgery, pharmacological therapy was started when the patients themselves showed a weight increase linked to a reduction in the control of food intake. The other 50 patients coming from other centers had a similar clinical history: at the time of weight regain after surgery or after insufficient weight loss, therapeutic interventions based on lifestyle changes were not effective as there was a loss of control of food intake. Our results showed a significant weight loss in all patients after a mean follow-up of 10.5 months. This important result of weight loss obtained after treatment with liraglutide in all subjects is probably due to the restoration of dietary control. We also observed that weight regain occurred regardless of the time elapsed after bariatric surgery. Some patients had weight regain even after a long-time, prompting us to consider the degree of weight regain and not the time elapsed between bariatric surgery and the start of liraglutide therapy.

Liraglutide therapy, which was initially continuous until weight goals were achieved, was in some subjects resumed when there was new weight regain or a new loss of dietary control.

In a limited number of patients, there was a partial or no response to liraglutide. In patients 36, 51, and 59 (see Table 2), the weight increase was slow and partial, and for this reason, according to the same patients, the treatment was prolonged from 12 to 16 months. In the other patients, where the weight regain was more marked, the therapy was interrupted at the same patient's request for lack of benefit on weight loss. These data confirm what it is currently known in the literature on patients not responding to liraglutide therapy.

Our study group in the past has been showed the possible role of pharmacological therapy in the management of weight loss after bariatric surgery [21, 22]. The use of different drugs as an adjunct to lifestyle modification has been reported in several small, uncontrolled, short-term prospective, and retrospective studies [23]. In a recent literature review, Redmond et al. report how GLP-1 agonists, as liraglutide and the once weekly formulation semaglutide, have been shown cardiovascular benefits in patients with diabetes and in older obese patients [23, 24]. We have been focused on non-diabetic obese patients, and in our subjects, the benefits of liraglutide treatment on weight loss after bariatric surgery occurred regardless of age and even in subjects younger than 60 years, as shown in Table 2.

In a retrospective study of Istfan et al. with a higher number of cases reported in the literature conducted from 2004 to 2015 on 1196 patients undergoing Roux-en-Y gastric bypass surgery, drug therapy with phentermine (34%) or phentermine–topiramate (44%) or topiramate alone (21%) was undertaken when weight regain was of 10% from the nadir achieved after surgery [19]. Similarly, in our study, liraglutide treatment was administered until patients achieved a minimum weight loss after bariatric surgery of 10–15% and was administered again when weight regain of 10–15% occurred.

Up to now, only a limited number of patients with weight regain after bariatric surgery have been able to take advantages of drug therapies, especially due to the cost of the same treatments that are charged to the patients.

In the literature, there are limited data on the use of liraglutide after bariatric surgery [6, 18, 20]. In particular, a retrospective study conducted in 2018 on 33 consecutive patients treated with liraglutide after any previous bariatric surgery showed a median percentage weight loss of 7.1% and a median BMI change of 4.7 kg/m^2^ after 28 weeks of therapy with no major adverse events [20]. Similarly, our data show the median change in BMI in all patients of 4.5 kg/m^2^ regardless of the surgery. According to the type of surgery, data were comparable (LGB: median change in BMI: LGB 4.7 kg/m^2^, LSG 4.5 kg/m^2^, and RYGB 4.7 kg/m^2^), indicating significant weight loss regardless of the bariatric surgery. Our results are similar to those reported by Wharton et al. [6] and are in contrast to those reported from the observational study of Suliman et al. where greater weight loss with liraglutide was obtained in patients previously treated with RYGB compared to LSG [25]. Finally, in line with the literature data, also in our study, there were no severe adverse events, while nausea and vomiting were the most frequent mild adverse events.

In conclusion, bariatric surgery is an effective option for obesity treatment, with a long-term weight maintenance. However, weight regain after bariatric surgeries can frequently occur and pharmacological therapy can be required. Our data confirm the efficacy and safety of pharmacological treatment with liraglutide in patients who experienced weight regain after bariatric surgery. Considering the long-term follow-up, patients should be followed up regularly and the pharmacological treatment should be adapted to the weight fluctuations observed during the clinical history.

What is already known on this subject?

To date, there are few data on the subject in the literature. Our retrospective real-life study confirms that the use of liraglutide after bariatric surgery is effective and safe, thus strengthening the results available to date in the literature.

What does this study add?

Our results not only confirm the few available results but highlight that liraglutide is effective regardless of the bariatric surgery performed.

## Data Availability

The datasets generated during and/or analyzed during the current study are available from the corresponding author on reasonable request.
